# Extended LUTS medication use following BPH surgical treatment: a US healthcare claims analysis

**DOI:** 10.1038/s41391-025-00953-0

**Published:** 2025-02-27

**Authors:** Steven Kaplan, Ronald P. Kaufman, Dean Elterman, Bilal Chughtai, Claus Roehrborn

**Affiliations:** 1 Icahn School of Medicine at Mount Sinai, Department of Urology, Mount Sinai, New York, NY USA; 2 Albany Medical College, Albany, NY USA; 3 University of Toronto, Toronto, ON Canada; 4 Northwell Health, Syosset, NY USA; 5 UT Southwestern Medical Center at Dallas, Frisco, TX USA

**Keywords:** Outcomes research, Prostatic diseases

## Abstract

**Background:**

Postoperative medication use is an important yet relatively unexplored element of the benign prostatic hyperplasia patient journey. We assessed and compared the percentage of patients who required medication postoperatively after the three most common BPH surgeries in the real world: transurethral resection of the prostate (TURP), photovaporization procedure with GreenLight Laser (PVP), and prostatic urethral lift (PUL) with the UroLift system.

**Methods:**

Within a random representative sample of US Medicare and commercial insurance claims, patients with at least one year of follow-up data available after an outpatient TURP, PVP, or PUL procedure were linked to pharmaceutical claims to elucidate rates of continuous and de novo use of alpha-blockers, 5-alpha reductase inhibitors, or combination medical therapy. Periods of interest were perioperative (use within three months postoperatively and not beyond) and one and five years postoperatively.

**Results:**

36 629 men diagnosed with BPH underwent outpatient TURP (*n* = 20 319), GreenLight PVP (*n* = 10 517) and PUL (*n* = 5 793) procedures within the claims dataset. The rate of medical therapy use through one year was lowest for PUL (4.1%) compared to TURP (6.2%) and PVP (6.6%), and was equivalent between procedures through five years (10.6% TURP, 10.4% PVP, and 10.3% PUL).

**Conclusions:**

Patients who undergo surgery to treat BPH may desire to discontinue or bypass BPH medications. However, these data demonstrated that approximately 10% of BPH patients used medication through five years postoperatively, regardless of which procedure they underwent.

## Introduction

Lower Urinary Tract Symptoms (LUTS) secondary to Benign Prostatic Hyperplasia (BPH) are estimated to affect more than 700 million men worldwide and approximately 42 million men in the US alone [1, 2]. The majority of patients are prescribed pharmaceutical treatment, while relatively few elect to undergo BPH procedures [3, 4]. Alpha-blockers and 5-alpha reductase inhibitors (5-ARIs) have long been at the forefront of pharmaceutical intervention. Numerous randomized controlled trials (RCTs) have established the safety and efficacy of these medications [5–8]; however, the high level of control in these studies prevents them from examining the overall effectiveness of these medications as they are implemented in healthcare systems. Studying patients longitudinally within healthcare systems has shown that patient adherence may be as low as 29% after one year [9–11]. Whether this low adherence is due to insufficient relief from symptoms, intolerance of the many known side effects, or a combination thereof remains to be studied.

The safety and efficacy of individual procedural treatments for BPH are typically supported by RCT data [12, 13]; however, relatively few studies have compared and contrasted between treatments in the real world. Recently, a healthcare utilization study following over 40,000 patients undergoing BPH procedures longitudinally for up to five years revealed interesting results regarding the effectiveness of implementation [14]. For the three most prevalent BPH procedures, transurethral resection of the prostate (TURP), laser photovaporization procedure (PVP), and prostatic urethral lift (PUL), observed complications and retreatment rates were not entirely as predicted by the RCT data. TURP, often cited as the most durable BPH procedure, demonstrated a retreatment rate of 5.3% at one year and 7% at five years. PVP retreatment rates were 5.3% and 8.9% at one and five years, and the rates for PUL were 5.9% and 11.6%, respectively. Complications, not unexpectedly, were higher for the more invasive cavitating procedures TURP and PVP.

RCT studies typically carefully control for postoperative medication usage, although reporting of usage rates has been inconsistent and may not be consistent with usage in common practice. Here, we report a five-year longitudinal study of BPH medication use after the three most prevalent BPH procedures, TURP, PVP, and PUL.

## Methods

### BPH index procedures

Transurethral resection of the prostate (TURP) removes obstructing prostate tissue by electrocautery cutting and irrigation removal of tissue sections. PVP employs a GreenLight™ Laser (Boston Scientific, Marlborough, Massachusetts, USA) to vaporize the prostate tissue. PUL using the UroLift™ System (Teleflex Inc., Pennsylvania, USA) reduces prostatic obstruction by deploying permanent implants in the prostatic fossa to hold the prostatic lumen open without removing tissue.

### Claims data

A random representative sample consisting of approximately 10% of all US BPH claims between 2015–2021 was acquired from IBM Watson Health Marketscan Research/Merative, a nationwide database with de-identified, individual-level claims from outpatient, inpatient, and prescription drug services for >230 million privately insured patients in the US. Payers included Medicare Administrative Contractors and commercial insurance. Only outpatient claims were used for BPH surgery utilization analyses. Within the claims data, the following medications were billed to insurance and identified via National Drug Code (NDC), then linked back to patients based on unique patient identifiers: Tamsulosin/Flomax, Alfuzosin/Uroxatal, Doxazosin/Cardura, Silodosin/Rapaflo, Terazosin/Hytrin, Finasteride/Proscar, Dutasteride/Avodart, and Jalyn/Combined Dutasteride and Tamsulosin (Supplemental Table 1). After filtering for each medication of interest, medications were grouped into one of three categories: Alpha-Blockers, 5-Alpha-Reductase Inhibitors, or Combination. Medication groups were not mutually exclusive, and patients could be included in zero to all groups; patients were not double-counted in total rates if they took more than one medication type. As the correlation between medication prescription and utilization can be unreliable, we defined medication usage as filling two consecutive prescriptions during the period of interest.

### Study population & definitions

The study population included males with BPH who underwent any of the three most prevalent BPH procedures (TURP, PVP, or PUL) between January 2015–June 2021. Diagnoses of BPH and relevant comorbidities were identified via International Classification of Diseases Clinical Modifications, 9th & 10th additions (ICD-9/10 CM). Index procedures were identified via Current Procedural Terminology (CPT) codes (Supplemental Table 2).

### Statistical analysis

Medication data were input as binary covariates in the multivariate models defined as 1 for patients who were on the medication of interest and 0 for patients not taking the drug during the periods of interest: preoperative, perioperative (up to three months post procedure), and through 1 and 5 years postoperative.

## Results

Within this representative sample of Medicare and commercial insurance claims from 2015-2021, 36,629 patients were diagnosed with BPH and underwent outpatient TURP (*n* = 20,319), PVP (*n* = 10,517), or PUL (*n* = 5 793), and had ≥1 year of follow-up data available. PUL patients at baseline were slightly younger (64.7 yrs) compared to TURP (66.9 yrs) and PVP (67.0 yrs) (Table 1). Urologically important comorbidities identified by ICD 9/10 diagnoses were low (most occurred at <5% in each group) and, with the exception of kidney disease, were moderately lower for both PVP and PUL than for TURP (Table 1).

**Table 1:** Baseline comorbidities by treatment.

Characteristic	*Statistics*	TURP	PVP	PUL
***n***		20319	10517	5793
**Age (years) at Treatment**	***Mean*** ***±*** ***SD***	66.9 ± 10.0	67.0 ± 10.1	64.7 ± 10.1
	*p value: vs TURP*		*p* = *0.2*	*p* < *0.0001*
**Hematuria**	***% of Subjects***	4.0%	2.6%	1.2%
	*p value: vs TURP*		*p* < *0.0001*	*p* < *0.0001*
**Kidney Disease**	***% of Subjects***	0.6%	0.6%	0.2%
	*p value: vs TURP*		*p* = *1.0*	*p* = *0.0004*
**Parkinson’s Disease**	***% of Subjects***	0.4%	0.2%	0.2%
	*p value: vs TURP*		*p* = *0.005*	*p* = *0.03*
**Prostate Cancer**	***% of Subjects***	2.6%	1.0%	0.6%
	*p value: vs TURP*		*p* < *0.0001*	*p* < *0.0001*
**Diabetes**	***% of Subjects***	8.0%	7.2%	5.6%
	*p value: vs TURP*		*p* = *0.01*	*p* < *0.0001*

### Perioperative medication usage

**Fig. 1:**
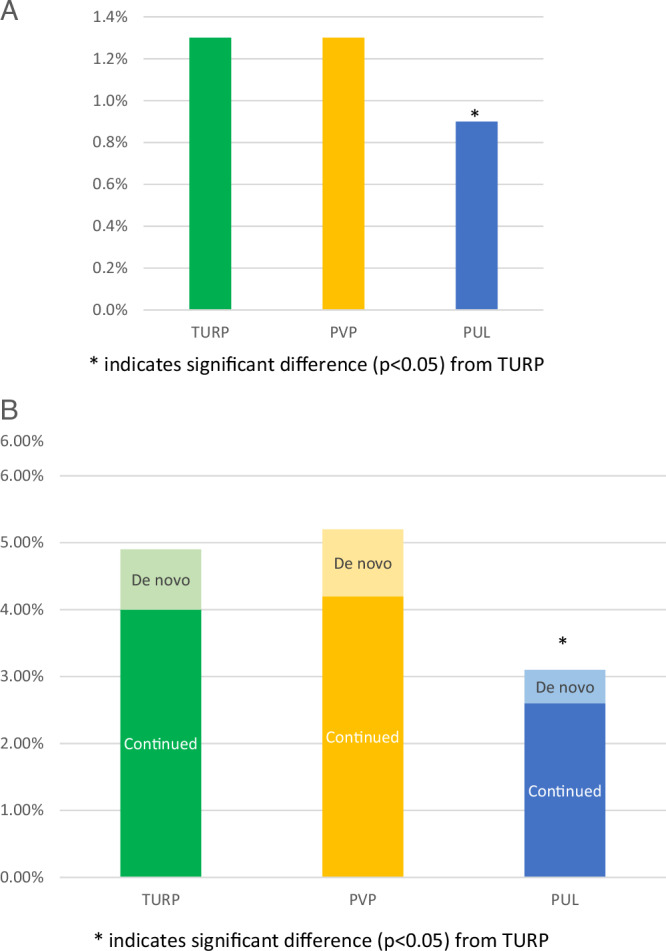
Rates of medication use through 1 Year. **A** Rates of perioperative medication use only; **B** Rates of medication use through 1 year, excluding perioperative use.

Defined as medication prescriptions within the first 3 months postoperatively and not at later time points, perioperative medical therapy rates were low for all three treatment groups. PVP and PUL perioperative medication usage was similar to TURP (PVP - 1.4%, TURP – 1.2%, PUL 0.9%) (Fig. 1A, Table 2). Alpha-blockers were the most utilized class of perioperative drug use, ranging from 71.1% of total postoperative medications for PVP to 90.7% for PUL (Supplemental Table 3).

**Table 2:** Medication therapy use through 1 and 5 years post-BPH procedures.

	TURP	PVP	PUL
**Perioperative Use Only**	1.2%	1.4%	0.9%
***p-value: vs TURP***		*p* = *0.4*	*p* = *0.06*
**1-year**			
**Continued use (med history prior to procedure)**	4.1%	4.3%	2.6%
***p-value: vs TURP***		*p* = *0.4*	*p* < *0.0001*
**De novo use (no med history)**	0.9%	1.0%	0.5%
***p-value: vs TURP***		*p* = *0.5*	*p* = *0.01*
**Total rate (perioperative + continued and de novo)**	6.2%	6.6%	4.1%
***p-value: vs TURP***		*p* = *0.05*	*p* < *0.0001*
**5-year**			
**Continued use (med history prior to procedure)**	7.2%	7.4%	8.4%
***p-value: vs TURP***		*p* = *0.7*	*p* = *0.5*
**De novo use (no med history)**	2.1%	1.7%	1.0%
***p-value: vs TURP***		*p* = *0.1*	*p* = *0.2*
**Total rate through 5 years (perioperative + continued and de novo)**	10.6%	10.4%	10.3%

### Medication usage through 1 year

Postoperative medication use was categorized into two groups: patients who had previously used medication before their surgery (“continued”), and those who had no recorded medication history prior to surgery (“de novo”). Continued medication, excluding perioperative use, after TURP was significantly higher compared to PUL (4.1% TURP vs. 2.6% PUL [*p* < 0.0001], and was similar to that after PVP (4.3% PVP [*p* = 0.4 vs TURP]; Fig. 1B, Table 2). Alpha-blockers were again the most utilized class of continued drugs in the 1-year time period (TURP 78.1%, PVP 78.5%, and PUL 84.9% of total med use after surgery) (Supplemental Table 3); 5ARI continued use through 1 year was 21.0% for TURP, 21.9% for PVP, and 14.4% for PUL out of total medications used (Supplemental Table 4).

**Fig. 2:**
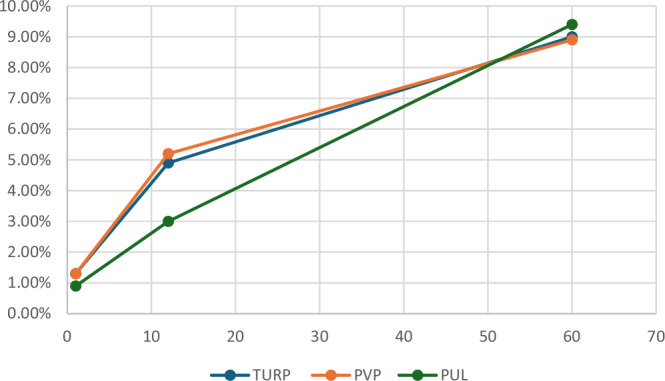
BPH Medication Use Through 5 Years.

De novo use was significantly higher in patients post-TURP (0.9%) compared to PUL (0.5%, *p* = 0.01), and was similar to PVP (1.0%, *p* = 0.5) (Fig. 2, Table 2). De novo use through one year mostly comprised alpha-blocker use (TURP 81.8%, PVP 81.2%, and PUL 76.7%) (Supplemental Table 3), and was 17.7%, 17.8%, and 23.3% for 5ARI use post-TURP, PVP, and PUL, respectively (Supplemental Table 4). Combination therapy occurred in <1% of patients through one year (Supplemental Table 5).

### Medication usage through five years

Medication use increased over five years in all surgery groups (Fig. 2). Continued medication use through five years occurred at statistically similar rates for TURP (7.2%) compared with PUL (8.4%, *p* = 0.5 vs TURP) and PVP (7.4%, *p* = 0.7 vs TURP) (Table 2). De novo medication use through 5 years was also statistically similar for TURP patients (2.1%) compared to PUL (1.0%, *p* = 0.2 vs TURP) and PVP (1.7%, *p* = 0.1 vs TURP).

When medication use was summed throughout the perioperative, 1-year, and 5-year time periods, the total medication use rates were equivalent among all three treatments (10.6% TURP, 10.4% PVP, and 10.3% PUL) (Table 2). Alpha-blocker use through 5 years made up 78.3% of total medication use for TURP patients, 77.4% for PVP, and 85.2% for PUL patients. 5-ARI use made up between 14.4% (PUL) - 21.9% (PVP) of total medication use (21.0% TURP). The proportion of combination use increased by five years postoperatively, with 1.3% for TURP, 0.8% for PVP, and 3.6% for PUL patients.

### Predictors of post-surgery medication use through 1 year

Logistic regression models were run to incorporate and account for differences in population variables, such as age and prevalence of comorbidities, to predict whether a patient will use (continued, de novo, or any) BPH medications for up to 1 year after surgery.

#### Logistic regression for any medication use through 1 year

Older age predicted any medication use through 1 year post-surgery. After controlling for this and all other relevant variables, those who underwent TURP or PVP were more likely to use medication, either continued or de novo, compared to PUL (OR 1.40 and OR 1.45, respectively) (Supplemental Table 6).

#### Logistic regression for continued medication use through 1 year

Continued medication use was predicted by older age and preoperative cystoscopy. PVP and TURP patients were more likely to continue medication through 1 year versus PUL patients (OR 1.28 and 1.24, respectively) (Supplemental Table 7).

#### Logistic regression for de novo medication use through 1 year

De novo medication use was predicted by older age and baseline comorbidities of stress incontinence and Parkinson’s disease. After accounting for the variables that conferred an increased risk of medication use and all other relevant variables, PVP and TURP patients were 53% and 43% more likely to use medication de novo post-surgery compared to PUL (Supplementary Table 8).

## Discussion

Healthcare utilization studies have strengths and weaknesses that must be acknowledged when interpreting findings. Tracking real medication usage versus prescription can be problematic; however, this study adds to the body of evidence by applying a robust metric for BPH medication usage and a large sample size. The primary strength of longitudinal database studies, such as this one, may be that they provide real-world occurrence rates for patients who elect these procedures. Retreatment, complications, and medication rates reflect the reality of how medicine is practiced today rather than the controlled dynamics of RCT studies, and may assist in educating patients and providers in better shared decision-making for BPH treatment.

The use of alpha-blockers or 5ARIs to treat LUTS after treatment with a BPH procedure is likely driven by a complex combination of clinical decisions. We found that perioperative use of these medications, presumably to “bridge” patients in the three months after surgery, was low, on the order of 1%, and higher for more invasive procedures than PUL. Overall, 10% of patients, regardless of treatment choice, were on BPH medication by 5 years, and 70–80% of these patients had restarted medication they were taking prior to surgery. As a database study, this study cannot elucidate why these medications were restarted or whether they helped symptoms, but it is reasonable to consider that they present a signal for continued bothersome LUTS post-surgery.

One possible interpretation of the increased rates of 1-year post-surgery medication use for TURP and PVP patients is that patients who are undergoing more invasive ablative procedures may have more advanced BPH disease than patients who elect the minimally invasive PUL. From this perspective, post-surgery medication use would demonstrate a difference in treatment populations rather than a lack of effectiveness of invasive procedures compared to PUL. We attempted to address this question with the data available within the claims database by analyzing and comparing available baseline data - that is, age and comorbidities - and thus gathered a panoramic view of the different patient populations. Indeed, we found that TURP and PVP patients were slightly older than PUL patients, with slightly elevated rates of comorbidities such as kidney disease, prostate cancer, and diabetes. By building a logistic regression model, we were able to effectively negate the effects of these factors on rates of post-surgery medication use through 1 year, as well as any other variable found to be a significant predictor of post-surgery medication use (e.g., stress incontinence and Parkinson’s disease), and found that TURP and PVP patients were still more likely to use post-surgery medication than PUL patients. Keeping these results in mind, it is still important to consider that we do not have data for pre-surgery symptom severity (e.g., IPSS, QoL, prostate volume), and that these factors could contribute to post-surgery medication use rates.

As with surgical retreatment reported in a prior study for this group [14], medication usage appears to be bimodal (Fig. 2). By one year post surgery, 5% to 6% of patients were on medication, followed by a slower annual increase to 5 years. A similar bimodal shape was observed for the retreatment of LUTS.

Comparing outcomes to those of RCT studies that rigorously reported medication usage showed an acceptable level of correlation. In a prospective, blinded RCT of TURP versus ablation, the reported continued medication use rate for TURP was 7.7% at 3 years [15], which is in line with our findings on TURP’s continued use (6.1% at 1 year and 10.2% at 5 years). A prospective study published in 2013 found an 8% medication use rate through 1 year for PVP [16]. In the LIFT pivotal trial for PUL, a 10.7% medication use rate was reported at 5 years, all of which were reported as alpha-blockers and/or 5ARI usage [13].

As longitudinal database studies require large populations, not all procedures utilized today could be examined. In recent years, steam thermal ablation has been introduced with limited increase in adoption; insufficient numbers existed in the healthcare database to report medication usage through five years. Similarly, resection using a high-velocity water jet and laser enucleation (i.e., HoLEP and TuLEP) are being increasingly adopted; however, low utilization at this point precludes proper analysis. Because the armamentarium of BPH procedures continues to evolve, it may be beneficial to periodically conduct longitudinal healthcare utilization studies to ensure a current understanding of real-world outcomes to best advise patients.

## Conclusions

Patients who undergo surgery to treat BPH may desire to discontinue their BPH medications or bypass them entirely. An interesting finding of this study is that at five years postoperatively, BPH medication usage centered around 10% of BPH surgery patients and appeared to be independent of treatment choice.

## Supplementary information

Supplemental Material Figure Legend

Supplemental Table 1

Supplemental Table 2

Supplemental Table 3

Supplemental Table 4

Supplemental Table 5

Supplemental Table 6

Supplemental Table 7

Supplemental Table 8

## Data Availability

The data that support the findings are available from Merative but restrictions apply to the availability of these data, which were used under license for the current study, and so are not publicly available. Data are however available from the authors upon reasonable request and with permission of Merative.
