# Correction: Extended LUTS medication use following BPH surgical treatment: a US healthcare claims analysis

**DOI:** 10.1038/s41391-025-00982-9

**Published:** 2025-05-16

**Authors:** Steven Kaplan, Ronald P. Kaufman, Dean Elterman, Bilal Chughtai, Claus Roehrborn

**Affiliations:** 1 Icahn School of Medicine at Mount Sinai, Department of Urology, Mount Sinai, New York, NY USA; 2 Albany Medical College, Albany, NY USA; 3 University of Toronto, Toronto, ON Canada; 4 Northwell Health, Syosset, NY USA; 5 UT Southwestern Medical Center at Dallas, Frisco, TX USA

**Keywords:** Outcomes research, Prostatic diseases

Correction to: *Prostate Cancer and Prostatic Diseases* 10.1038/s41391-025-00953-0, published online 27 February 2025

The article “Extended LUTS medication use following BPH surgical treatment: a US healthcare claims analysis”, written by Steven Kaplan, Ronald P. Kaufman Jr, Dean Elterman, Bilal Chughtai and Claus Roehrborn, was originally published under exclusive license to The Author(s), under exclusive licence to Springer Nature Limited 2025. As a result of the subsequent decision to publish the article under the open access model, the article’s copyright notice was changed on 15 April, 2025 to © The Author(s) 2025 and the article is now distributed under a Creative Commons Attribution 4.0 International License, which permits use, sharing, adaptation, distribution and reproduction in any medium or format, as long as you give appropriate credit to the original author(s) and the source, provide a link to the Creative Commons licence, and indicate if changes were made. The images or other third party material in this article are included in the article’s Creative Commons licence, unless indicated otherwise in a credit line to the material. If material is not included in the article’s Creative Commons licence and your intended use is not permitted by statutory regulation or exceeds the permitted use, you will need to obtain permission directly from the copyright holder. To view a copy of this licence, visit http://creativecommons.org/licenses/by/4.0/.

