# Supplementary material for: Extended LUTS medication use following BPH surgical treatment: a US healthcare claims analysis
Source: Prostate Cancer Prostatic Dis. 2025 Feb 27;28(4):913–7. doi: 10.1038/s41391-025-00953-0 (PMC12643914; doi:10.1038/s41391-025-00953-0)
Supplement: Supplementary file 1 — Supplemental Material Figure Legend [file 41391_2025_953_MOESM1_ESM.docx]

Supplemental Table 1. List of NDC codes used for BPH medications

Supplemental Table 2. List of CPT codes used for index procedures

Supplemental Table 3. Rates of alpha-blocker use through 1 and 5 years post-BPH procedures

Supplemental Table 4. Rates of 5ARI use through 1 and 5 years post-BPH procedures

Supplemental Table 5. Rates of combination use through 1 and 5 years post-BPH procedures

Supplemental Table 6. Logistic model of factors increasing the likelihood of a patient using any medication post-procedure

Supplemental Table 7. Logistic model of factors increasing the likelihood of a patient having continued use of medication post-procedure

Supplemental Table 8. Logistic model of factors increasing the likelihood of a patient having de novo use of medication post-procedure
