# Supplementary material for: Extended LUTS medication use following BPH surgical treatment: a US healthcare claims analysis
Source: Prostate Cancer Prostatic Dis. 2025 Feb 27;28(4):913–7. doi: 10.1038/s41391-025-00953-0 (PMC12643914; doi:10.1038/s41391-025-00953-0)
Supplement: Supplementary file 4 — Supplemental Table 3 [file 41391_2025_953_MOESM4_ESM.pptx]

## Slide 1
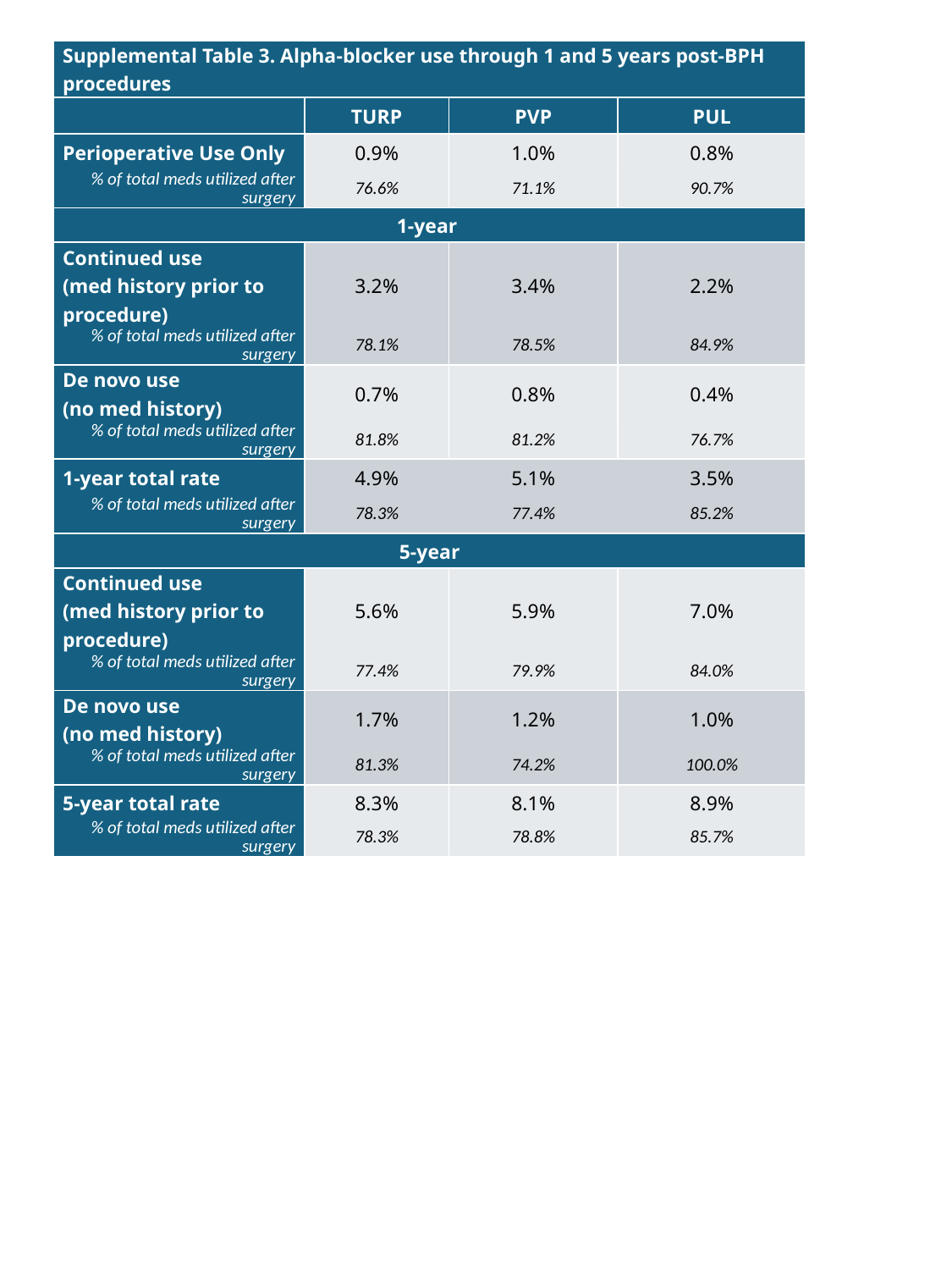

| Supplemental Table 3. Alpha-blocker use through 1 and 5 years post-BPH procedures | | | |
| --- | --- | --- | --- |
| | TURP | PVP | PUL |
| Perioperative Use Only | 0.9% | 1.0% | 0.8% |
| % of total meds utilized after surgery | 76.6% | 71.1% | 90.7% |
| 1-year | | | |
| Continued use (med history prior to procedure) | 3.2% | 3.4% | 2.2% |
| % of total meds utilized after surgery | 78.1% | 78.5% | 84.9% |
| De novo use (no med history) | 0.7% | 0.8% | 0.4% |
| % of total meds utilized after surgery | 81.8% | 81.2% | 76.7% |
| 1-year total rate | 4.9% | 5.1% | 3.5% |
| % of total meds utilized after surgery | 78.3% | 77.4% | 85.2% |
| 5-year | | | |
| Continued use (med history prior to procedure) | 5.6% | 5.9% | 7.0% |
| % of total meds utilized after surgery | 77.4% | 79.9% | 84.0% |
| De novo use (no med history) | 1.7% | 1.2% | 1.0% |
| % of total meds utilized after surgery | 81.3% | 74.2% | 100.0% |
| 5-year total rate | 8.3% | 8.1% | 8.9% |
| % of total meds utilized after surgery | 78.3% | 78.8% | 85.7% |
