# Supplementary material for: Extended LUTS medication use following BPH surgical treatment: a US healthcare claims analysis
Source: Prostate Cancer Prostatic Dis. 2025 Feb 27;28(4):913–7. doi: 10.1038/s41391-025-00953-0 (PMC12643914; doi:10.1038/s41391-025-00953-0)
Supplement: Supplementary file 5 — Supplemental Table 4 [file 41391_2025_953_MOESM5_ESM.pptx]

## Slide 1
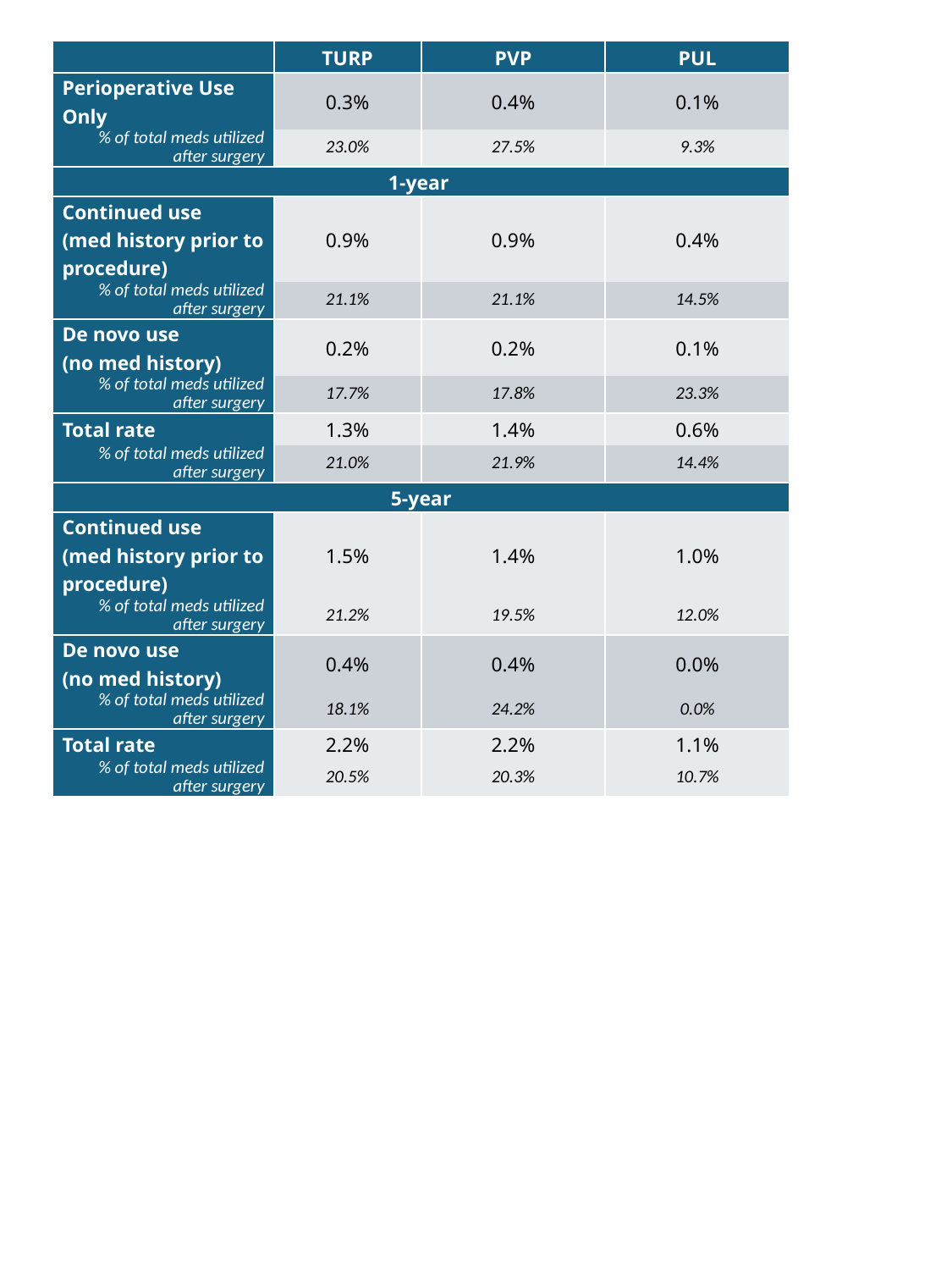

| | TURP | PVP | PUL |
| --- | --- | --- | --- |
| Perioperative Use Only | 0.3% | 0.4% | 0.1% |
| % of total meds utilized after surgery | 23.0% | 27.5% | 9.3% |
| 1-year | | | |
| Continued use (med history prior to procedure) | 0.9% | 0.9% | 0.4% |
| % of total meds utilized after surgery | 21.1% | 21.1% | 14.5% |
| De novo use (no med history) | 0.2% | 0.2% | 0.1% |
| % of total meds utilized after surgery | 17.7% | 17.8% | 23.3% |
| Total rate | 1.3% | 1.4% | 0.6% |
| % of total meds utilized after surgery | 21.0% | 21.9% | 14.4% |
| 5-year | | | |
| Continued use (med history prior to procedure) | 1.5% | 1.4% | 1.0% |
| % of total meds utilized after surgery | 21.2% | 19.5% | 12.0% |
| De novo use (no med history) | 0.4% | 0.4% | 0.0% |
| % of total meds utilized after surgery | 18.1% | 24.2% | 0.0% |
| Total rate | 2.2% | 2.2% | 1.1% |
| % of total meds utilized after surgery | 20.5% | 20.3% | 10.7% |
