# Supplementary material for: Extended LUTS medication use following BPH surgical treatment: a US healthcare claims analysis
Source: Prostate Cancer Prostatic Dis. 2025 Feb 27;28(4):913–7. doi: 10.1038/s41391-025-00953-0 (PMC12643914; doi:10.1038/s41391-025-00953-0)
Supplement: Supplementary file 6 — Supplemental Table 5 [file 41391_2025_953_MOESM6_ESM.pptx]

## Slide 1
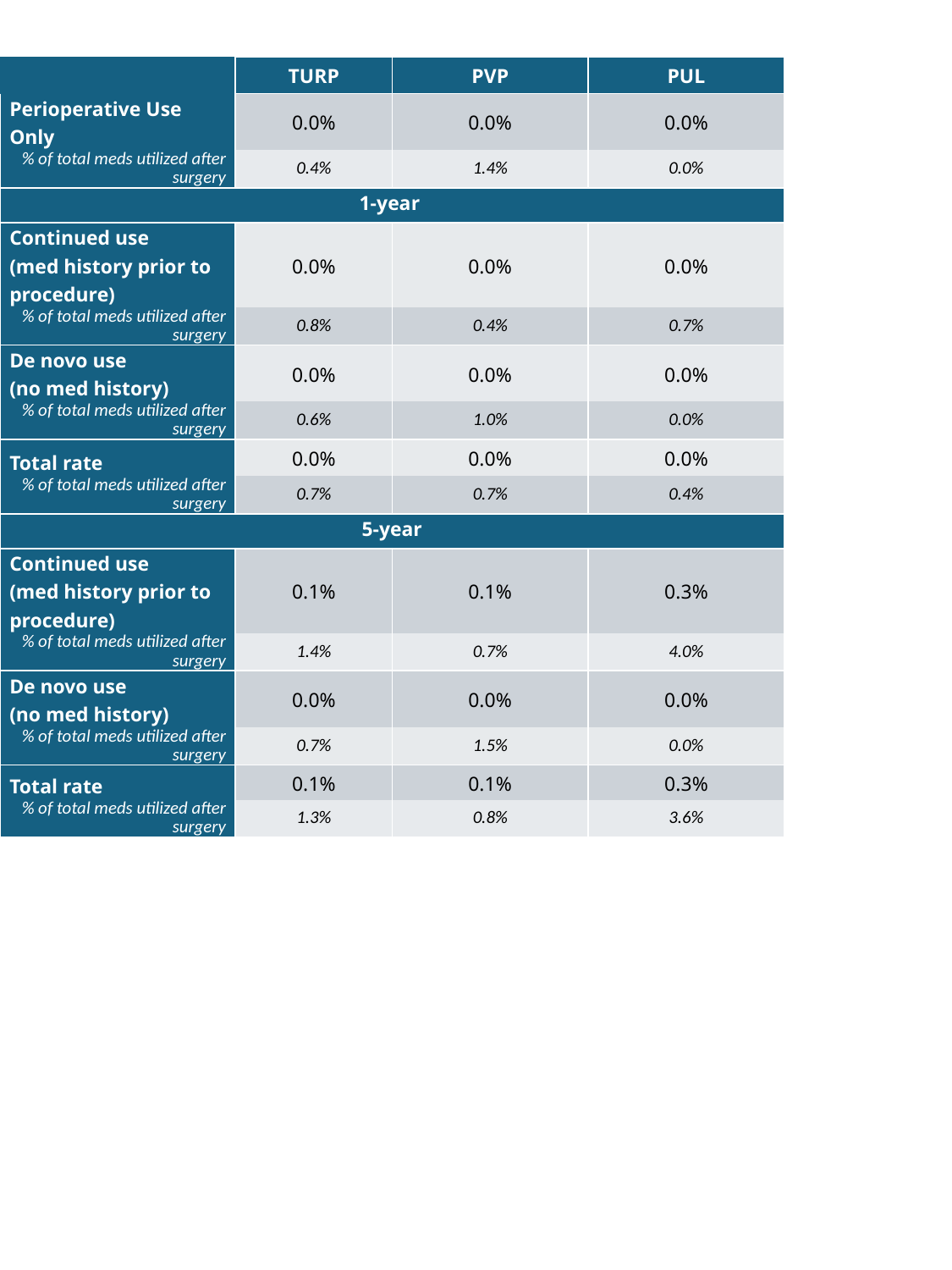

| | TURP | PVP | PUL |
| --- | --- | --- | --- |
| Perioperative Use Only | 0.0% | 0.0% | 0.0% |
| % of total meds utilized after surgery | 0.4% | 1.4% | 0.0% |
| 1-year | | | |
| Continued use (med history prior to procedure) | 0.0% | 0.0% | 0.0% |
| % of total meds utilized after surgery | 0.8% | 0.4% | 0.7% |
| De novo use (no med history) | 0.0% | 0.0% | 0.0% |
| % of total meds utilized after surgery | 0.6% | 1.0% | 0.0% |
| Total rate | 0.0% | 0.0% | 0.0% |
| % of total meds utilized after surgery | 0.7% | 0.7% | 0.4% |
| 5-year | | | |
| Continued use (med history prior to procedure) | 0.1% | 0.1% | 0.3% |
| % of total meds utilized after surgery | 1.4% | 0.7% | 4.0% |
| De novo use (no med history) | 0.0% | 0.0% | 0.0% |
| % of total meds utilized after surgery | 0.7% | 1.5% | 0.0% |
| Total rate | 0.1% | 0.1% | 0.3% |
| % of total meds utilized after surgery | 1.3% | 0.8% | 3.6% |
