# Supplementary material for: Extended LUTS medication use following BPH surgical treatment: a US healthcare claims analysis
Source: Prostate Cancer Prostatic Dis. 2025 Feb 27;28(4):913–7. doi: 10.1038/s41391-025-00953-0 (PMC12643914; doi:10.1038/s41391-025-00953-0)
Supplement: Supplementary file 7 — Supplemental Table 6 [file 41391_2025_953_MOESM7_ESM.pptx]

## Slide 1
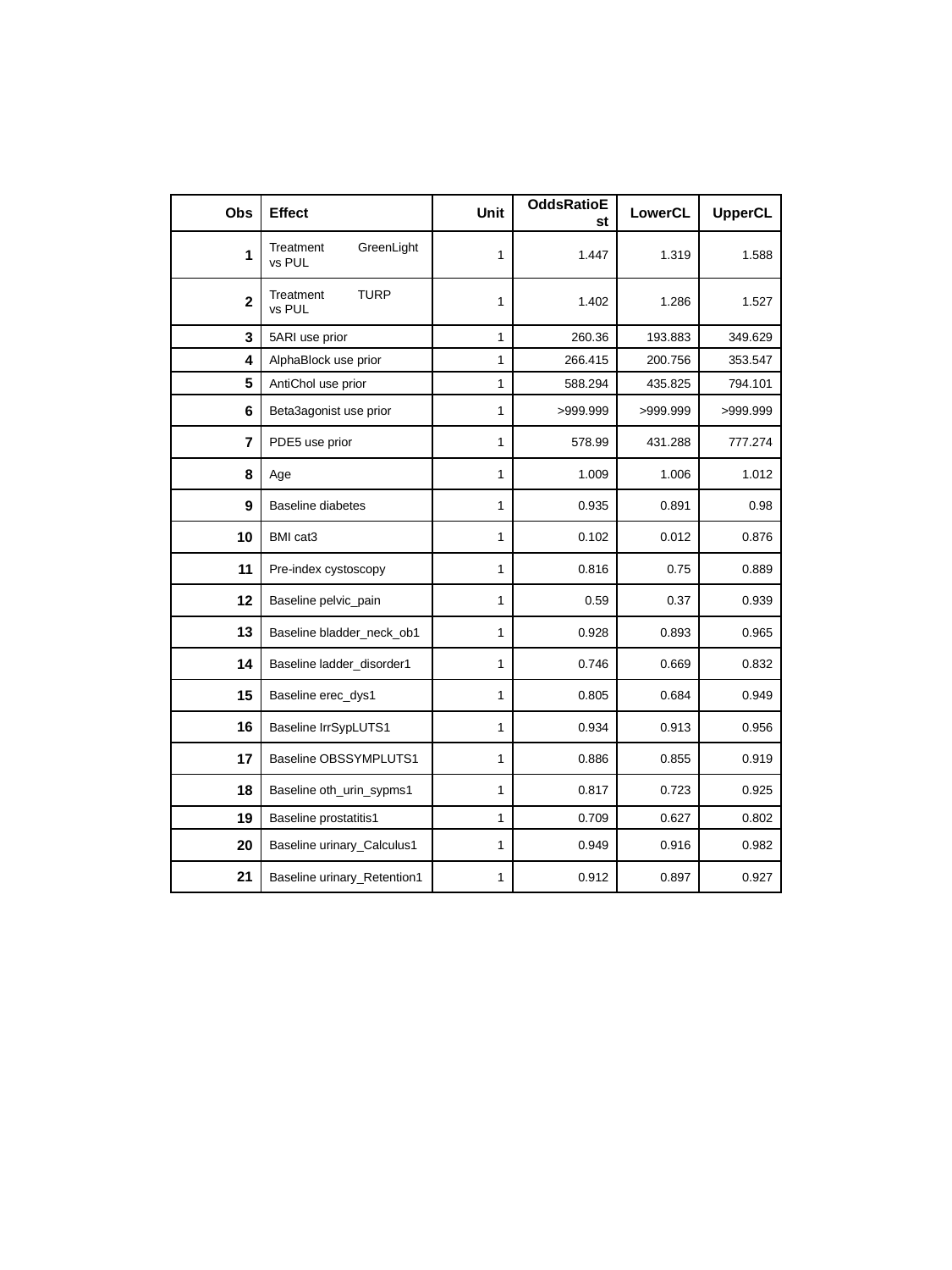

| | | | | | |
| --- | --- | --- | --- | --- | --- |
| | | | | | |
| | | | | | |
| Obs | Effect | Unit | OddsRatioEst | LowerCL | UpperCL |
| 1 | Treatment GreenLight vs PUL | 1 | 1.447 | 1.319 | 1.588 |
| 2 | Treatment TURP vs PUL | 1 | 1.402 | 1.286 | 1.527 |
| 3 | 5ARI use prior | 1 | 260.36 | 193.883 | 349.629 |
| 4 | AlphaBlock use prior | 1 | 266.415 | 200.756 | 353.547 |
| 5 | AntiChol use prior | 1 | 588.294 | 435.825 | 794.101 |
| 6 | Beta3agonist use prior | 1 | >999.999 | >999.999 | >999.999 |
| 7 | PDE5 use prior | 1 | 578.99 | 431.288 | 777.274 |
| 8 | Age | 1 | 1.009 | 1.006 | 1.012 |
| 9 | Baseline diabetes | 1 | 0.935 | 0.891 | 0.98 |
| 10 | BMI cat3 | 1 | 0.102 | 0.012 | 0.876 |
| 11 | Pre-index cystoscopy | 1 | 0.816 | 0.75 | 0.889 |
| 12 | Baseline pelvic\_pain | 1 | 0.59 | 0.37 | 0.939 |
| 13 | Baseline bladder\_neck\_ob1 | 1 | 0.928 | 0.893 | 0.965 |
| 14 | Baseline ladder\_disorder1 | 1 | 0.746 | 0.669 | 0.832 |
| 15 | Baseline erec\_dys1 | 1 | 0.805 | 0.684 | 0.949 |
| 16 | Baseline IrrSypLUTS1 | 1 | 0.934 | 0.913 | 0.956 |
| 17 | Baseline OBSSYMPLUTS1 | 1 | 0.886 | 0.855 | 0.919 |
| 18 | Baseline oth\_urin\_sypms1 | 1 | 0.817 | 0.723 | 0.925 |
| 19 | Baseline prostatitis1 | 1 | 0.709 | 0.627 | 0.802 |
| 20 | Baseline urinary\_Calculus1 | 1 | 0.949 | 0.916 | 0.982 |
| 21 | Baseline urinary\_Retention1 | 1 | 0.912 | 0.897 | 0.927 |
