# Supplementary material for: Extended LUTS medication use following BPH surgical treatment: a US healthcare claims analysis
Source: Prostate Cancer Prostatic Dis. 2025 Feb 27;28(4):913–7. doi: 10.1038/s41391-025-00953-0 (PMC12643914; doi:10.1038/s41391-025-00953-0)
Supplement: Supplementary file 8 — Supplemental Table 7 [file 41391_2025_953_MOESM8_ESM.pptx]

## Slide 1
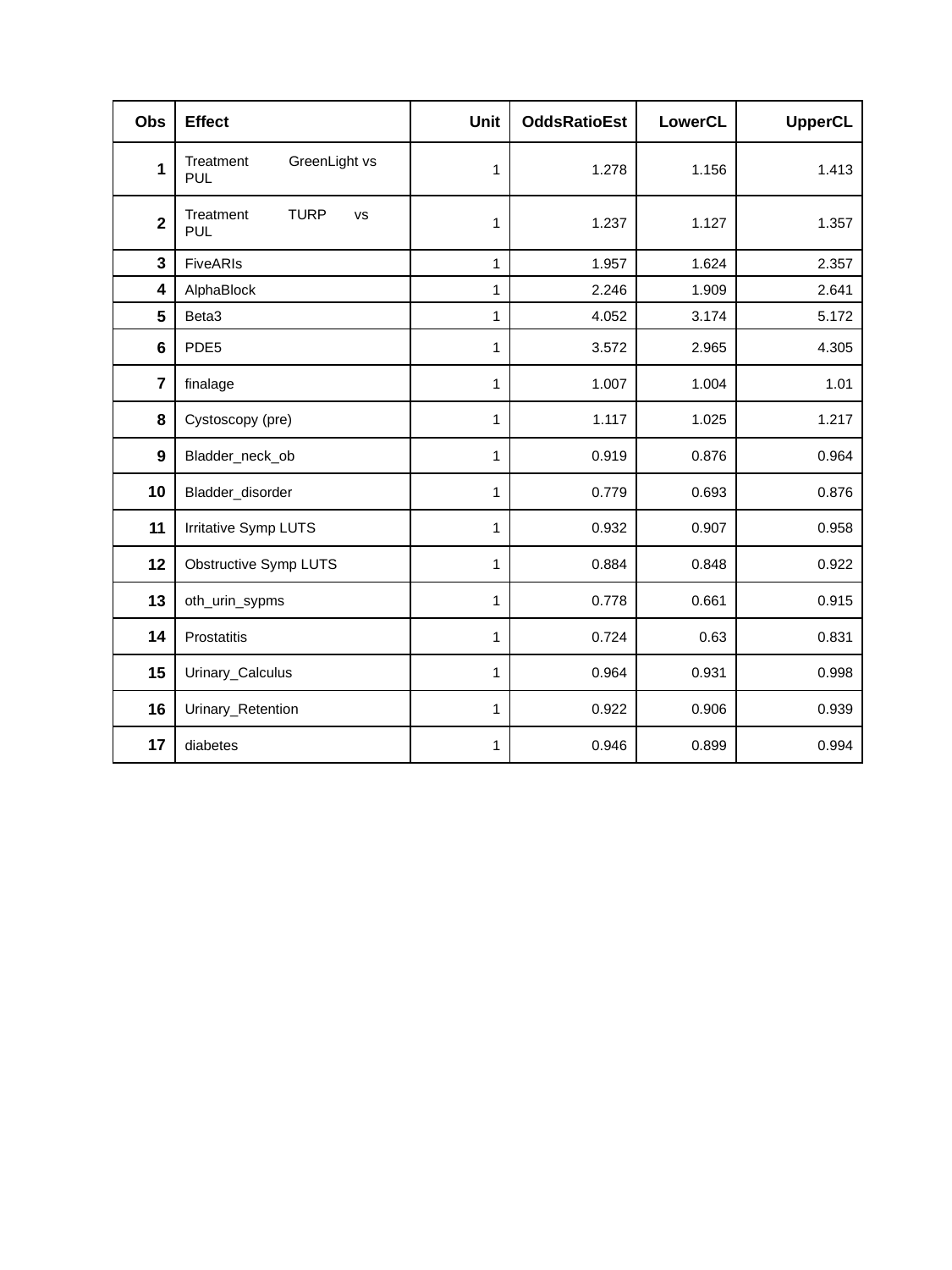

| | | | | | |
| --- | --- | --- | --- | --- | --- |
| Obs | Effect | Unit | OddsRatioEst | LowerCL | UpperCL |
| 1 | Treatment GreenLight vs PUL | 1 | 1.278 | 1.156 | 1.413 |
| 2 | Treatment TURP vs PUL | 1 | 1.237 | 1.127 | 1.357 |
| 3 | FiveARIs | 1 | 1.957 | 1.624 | 2.357 |
| 4 | AlphaBlock | 1 | 2.246 | 1.909 | 2.641 |
| 5 | Beta3 | 1 | 4.052 | 3.174 | 5.172 |
| 6 | PDE5 | 1 | 3.572 | 2.965 | 4.305 |
| 7 | finalage | 1 | 1.007 | 1.004 | 1.01 |
| 8 | Cystoscopy (pre) | 1 | 1.117 | 1.025 | 1.217 |
| 9 | Bladder\_neck\_ob | 1 | 0.919 | 0.876 | 0.964 |
| 10 | Bladder\_disorder | 1 | 0.779 | 0.693 | 0.876 |
| 11 | Irritative Symp LUTS | 1 | 0.932 | 0.907 | 0.958 |
| 12 | Obstructive Symp LUTS | 1 | 0.884 | 0.848 | 0.922 |
| 13 | oth\_urin\_sypms | 1 | 0.778 | 0.661 | 0.915 |
| 14 | Prostatitis | 1 | 0.724 | 0.63 | 0.831 |
| 15 | Urinary\_Calculus | 1 | 0.964 | 0.931 | 0.998 |
| 16 | Urinary\_Retention | 1 | 0.922 | 0.906 | 0.939 |
| 17 | diabetes | 1 | 0.946 | 0.899 | 0.994 |
