# Supplementary material for: Extended LUTS medication use following BPH surgical treatment: a US healthcare claims analysis
Source: Prostate Cancer Prostatic Dis. 2025 Feb 27;28(4):913–7. doi: 10.1038/s41391-025-00953-0 (PMC12643914; doi:10.1038/s41391-025-00953-0)
Supplement: Supplementary file 9 — Supplemental Table 8 [file 41391_2025_953_MOESM9_ESM.pptx]

## Slide 1
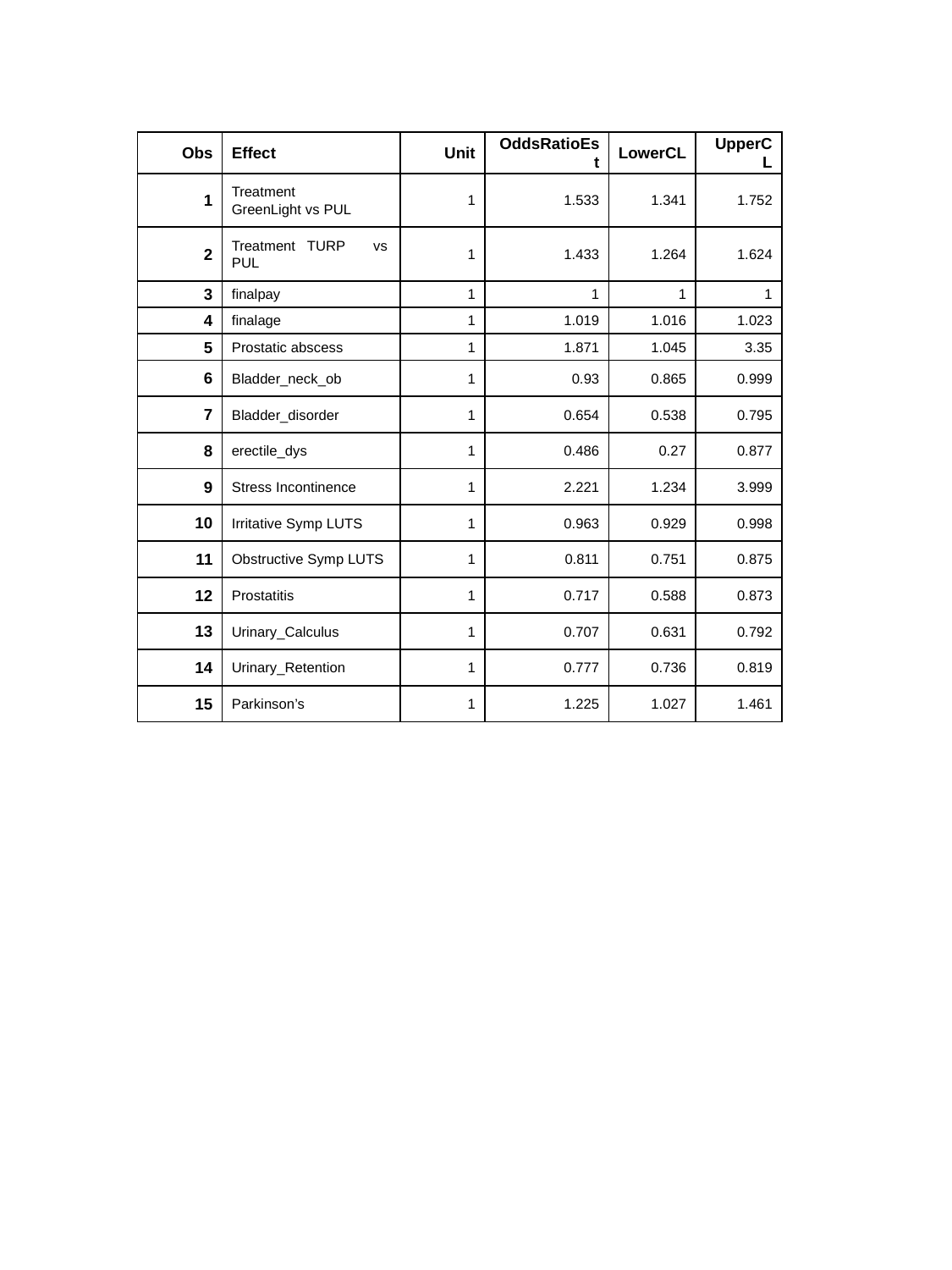

| | | | | | |
| --- | --- | --- | --- | --- | --- |
| Obs | Effect | Unit | OddsRatioEst | LowerCL | UpperCL |
| 1 | Treatment GreenLight vs PUL | 1 | 1.533 | 1.341 | 1.752 |
| 2 | Treatment TURP vs PUL | 1 | 1.433 | 1.264 | 1.624 |
| 3 | finalpay | 1 | 1 | 1 | 1 |
| 4 | finalage | 1 | 1.019 | 1.016 | 1.023 |
| 5 | Prostatic abscess | 1 | 1.871 | 1.045 | 3.35 |
| 6 | Bladder\_neck\_ob | 1 | 0.93 | 0.865 | 0.999 |
| 7 | Bladder\_disorder | 1 | 0.654 | 0.538 | 0.795 |
| 8 | erectile\_dys | 1 | 0.486 | 0.27 | 0.877 |
| 9 | Stress Incontinence | 1 | 2.221 | 1.234 | 3.999 |
| 10 | Irritative Symp LUTS | 1 | 0.963 | 0.929 | 0.998 |
| 11 | Obstructive Symp LUTS | 1 | 0.811 | 0.751 | 0.875 |
| 12 | Prostatitis | 1 | 0.717 | 0.588 | 0.873 |
| 13 | Urinary\_Calculus | 1 | 0.707 | 0.631 | 0.792 |
| 14 | Urinary\_Retention | 1 | 0.777 | 0.736 | 0.819 |
| 15 | Parkinson’s | 1 | 1.225 | 1.027 | 1.461 |
